# The Effect of Microwave Radiation on the Self-Healing Performance of Asphalt Mixtures with Steel Slag Aggregates and Steel Fibers

**DOI:** 10.3390/ma16103712

**Published:** 2023-05-13

**Authors:** Carlos D. A. Loureiro, Hugo M. R. D. Silva, Joel R. M. Oliveira, Nuno L. S. Costa, Carlos A. O. Palha

**Affiliations:** 1 ISISE—Institute for Sustainability and Innovation in Structural Engineering, Department of Civil Engineering, University of Minho, 4800-058 Guimaraes, Portugal; id9629@alunos.uminho.pt (C.D.A.L.); joliveira@civil.uminho.pt (J.R.M.O.); a79917@alunos.uminho.pt (N.L.S.C.); 2 Department of Civil Engineering, University of Minho, 4800-058 Guimaraes, Portugal; cpalha@civil.uminho.pt

**Keywords:** self-healing, steel slag aggregates, steel wool fibers, asphalt mixtures, microwave radiation heating, self-healing assessment

## Abstract

Self-healing in asphalt mixtures is a property that can be enhanced by external heating, which causes a thermal expansion that increases the flow of bitumen with reduced viscosity through the cracks. Therefore, this study aims to evaluate the effects of microwave heating on the self-healing performance of three asphalt mixtures: (1) conventional, (2) with steel wool fibers (SWF), and (3) with steel slag aggregates (SSA) and SWF. After evaluating the microwave heating capacity of the three asphalt mixtures with a thermographic camera, their self-healing performance was determined with fracture or fatigue tests and microwave heating recovery cycles. The results demonstrated that the mixtures with SSA and SWF promoted higher heating temperatures and presented the best self-healing capacity during the semicircular bending test and heating cycles, with significant strength recovery after a total fracture. In contrast, the mixtures without SSA presented inferior fracture results. Both the conventional mixture and that containing SSA and SWF presented high healing indexes after the four-point bending fatigue test and heating cycles, with a fatigue life recovery of around 150% after applying two healing cycles. Therefore, the conclusion is that SSA greatly influences the self-healing performance of asphalt mixtures after microwave radiation heating.

## 1. Introduction

Industrial and technological development, combined with demands for a better quality of life, has increased the consumption of material resources, making it impossible for the planet to continue on this path. Thus, society has become aware that resources are finite and that reducing their consumption and use is imperative.

The construction industry is one of Europe’s largest and most active economic sectors. However, it is also responsible for a significant environmental impact due to its high energy consumption, how it develops, uses, distributes, and disposes of virgin resources, and produces excessive greenhouse gas emissions [1,2].

More specifically, the road infrastructure sector damages the environment and the planet’s sustainability, from transporting goods and people to constructing pavements. Thus, changing this sector’s current design, management, and monitoring paradigm is essential. This sector must develop policies to achieve sustainable development objectives effectively, creating innovative processes aimed at reducing CO_2_ emissions, reducing the use of virgin materials, and reusing waste in construction while ensuring positive effects from an economic, technical, and environmental point of view [3,4].

Based on this line of action, this paper aimed to study whether the incorporation of industrial by-products, steel slag aggregates, and steel fibers, steel wool fibers, promotes the enhancement of the self-healing of asphalt mixtures when these are subjected to heating by microwave radiation. This study is expected to contribute to a more sustainable paving area, promoting the increase of pavement lifetime and minimizing the consumption of virgin materials in pavement rehabilitation/conservation works.

Given the lack of previous studies on the self-healing performance of asphalt mixtures incorporating high amounts of by-products, this study will undoubtedly contribute to increasing the scientific community’s knowledge of this research topic.

## 2. Literature Review

Sustainability and circular economy principles are increasingly present in the decision making of governmental agencies due to society’s growing concern with the planet’s environment, which will bring profound transformations to the road pavement industry. The environmental and economic costs associated with constructing new road infrastructures or the conservation/rehabilitation of existing pavements are considered excessive and inadequate. Consequently, there is an urgent need to develop innovative paving solutions to change the current consumption paradigm. From this perspective, this article aims to assess solutions that simultaneously incorporate industrial by-products (steel slag) in the production of asphalt mixtures and promote an increase in the pavement’s service life through self-healing.

Steel slag aggregates (SSA) originate from the crude steel production process’s by-product, and these could be used in several industrial sectors: road infrastructures, cement, concrete, and additives [5]. Correia et al. [6] reported that the steel industry produces about 11 to 15% black slag and 2 to 3% white slag (for this slag, its use in construction is discarded). According to the World Steel Association [7], in 2020, crude steel production was about 1900 million tons, so it was possible to extract about 200–285 million tons of steel slag. Therefore, given the current environmental concerns, using steel slag to produce aggregates for pavement construction is an excellent contribution to the planet’s sustainability.

In a review paper [8], the authors concluded that several properties of steel slag aggregates (e.g., roughness, shape, angularity, hardness, polishing resistance, and wear resistance) make them suitable for use in asphalt mixtures. In brief, the SSAs have a low presence of fines, a high sand equivalent, a good shape with high angularity, rough texture, a high density (3.2–3.8 g/cm^3^), and high porosity and water absorption [9]. In another study, the authors revealed that the SSAs are a by-product with great potential to be used as aggregates in asphalt mixtures due to their excellent wearing and polishing resistance and good affinity with bitumen. Moreover, based on the results obtained, the authors concluded that the mixtures showed excellent performance evaluated through water sensitivity and rutting resistance tests, surpassing the results obtained for conventional asphalt mixtures with identical compositions produced with natural aggregates [10].

Even though asphalt pavements have many advantages (cost efficiency, noise reduction, comfortable driving experience), asphalt concrete undergoes functional and structural degradation after a few years of service, mainly due to traffic and weather conditions. As the asphalt binder ages, it becomes more brittle, leading initially to micro-cracks and then to macro-cracks, which lead to pavement performance failure, both from a structural and functional point of view [11]. So, one of the solutions to increase the durability of infrastructures is to use high-quality materials with self-healing capabilities.

In recent years, self-healing has expanded from chemistry to engineering. This phenomenon has multiple and unique characteristics that make it a promising approach to increasing the durability of asphalt pavements by regenerating micro-cracks and recovering the initial performance without reconstructing the pavement [12].

This behavior is only possible since bitumen is a viscoelastic fluid with a relatively long surface wetting and diffusion capability, exhibiting the behavior of a self-healing material [13]. Therefore, bitumen can close micro-cracks and restore the asphalt’s mixture strength and resistance. However, the mechanism that causes self-healing is not yet fully understood. There is still neither a single definition nor a standardized method for predicting the self-healing capacity of asphalt materials. In these materials, some internal factors influence the self-healing performance: molecular composition, structure, asphalt movement and diffusion, asphalt viscoelasticity, and thixotropy. Externally, factors such as humidity, temperature, induction conditions, and induction duration are also influential in the performance of this phenomenon [14]. Thus, to better understand this behavior, this article aims to study the influence of microwave radiation heating to enhance self-healing in sustainable asphalt mixtures.

Electromagnetic induction and microwave radiation are the processes most commonly used to promote heating asphalt mixtures to enhance their self-healing effectiveness. The induction heating method combines electromagnetic induction and Joule heating effects. Faraday’s law states that an electromotive force is induced in a conductive material when an alternating magnetic field is present [15]. Therefore, electrically conductive asphalt materials can be developed based on that principle, but the key challenge lies in effectively improving their conductivity without reducing their mechanical performance on the pavement. There are different additives to incorporate into the mix design of asphalt mixtures. However, introducing metallic materials (e.g., steel fiber, magnetite, carbonyl iron powder, and steel slag) is the best option. As a result, heating by electromagnetic induction is impossible without the presence of a conductive material [15].

Meanwhile, the working principle of microwave radiation, called dielectric heating, refers to heating a dielectric medium by the electric field component of high-frequency radiation [15]. Interestingly, microwave radiation heating does not rely on conductive materials and works directly on the dielectric material. Thus, this technique can heat conventional asphalt mixtures without any conductive materials, although using conductive additives can accelerate heating [15].

Previous studies have focused on incorporating conductible materials into asphalt mixtures to speed up the heating of mixtures subjected to electromagnetic induction or microwave radiation. The heating efficiency depends on many factors, including the conductivity and other characteristics (e.g., type, diameter, length, and percentage) of the additives/fibers added to the mixture, their spatial distribution, and the type of aggregates and fillers used in the mixtures. Theoretically, it is feasible to repair cracks in asphalt concrete by adding conducting fibers, improving its conductivity, and heating the mixture with induction energy to increase its self-healing rate [16]. García et al. [17] defended that the amount of heat generated in induction heating depends mainly on the radius of the fibers rather than on their volume in the mixture. According to Norambuena-Contreras and Garcia [18], crack healing of asphalt mixtures by microwave heating can be achieved by adding metallic fibers to the asphalt mixture composition. These fibers can increase the heating rates of asphalt mixtures since they can absorb and conduct more thermal energy than the other components of the mixture (i.e., aggregates and bitumen). The authors concluded that a higher fiber content contributes to fiber clusters and mix oxidation.

Incorporating SSA in asphalt mixtures, besides reducing the consumption of natural aggregates, also contributes to enhancing the heating of asphalt mixtures through induction heating systems due to their chemical composition. Steel slag is a desirable low-cost absorber to trigger microwave heating with excellent magnetic properties [19]. In another study, the authors concluded that replacing 30% natural aggregates with steel slag aggregates provides better healing results and helps the mixture improve the load–displacement relationship with higher ductile behavior [20]. Wan et al. [21] studied how the composition and size of steel slag aggregates affect the asphalt mixtures’ induction heating efficiency. This paper investigated the steel slag’s heating efficiency based on its components, size, and iron element content. The authors concluded that the steel slags could be heated by induction and that iron and Fe_3_O_4_ were the active components. Besides, steel slag aggregates with higher iron content generally had higher heating efficiency. Li et al. [22] studied the feasibility of replacing limestone filler with steel slag filler to enhance the heat release and self-healing properties of asphalt mastics. The authors concluded that the steel slag filler-based asphalt mastics showed better healing rates than the limestone filler ones because the steel slag filler could convert the energy of microwave irradiation into more thermal energy.

Evaluating self-healing effectiveness is one of the most critical tasks in this type of research. Several specific tools, methods, and tests have been applied to quantify the success of self-healing in different asphalt materials (i.e., bitumen, mastics, and asphalt mixtures), which depend on the type of material studied. In particular, the semicircular bending (SCB) fracture test and the four-point bending fatigue test are the principal tests used for measuring the self-healing efficiency of asphalt mixtures [18,23,24].

In this type of scientific study, there are naturally some limitations. The main limitation is its applicability in a practical context, as microwave radiation heating is distributed more or less uniformly over the whole surface area of the specimen. In contrast, the heating of actual road pavements can only be done on the pavement’s surface course. Thus, comparative studies should be made with other heating methods, e.g., electromagnetic induction, to see which is the most efficient in a laboratory and practical context. Furthermore, in the future, an environmental and economic study of the life cycle of an asphalt mixture with this heating method should be carried out to validate its feasibility.

## 3. Materials and Methods

### 3.1. Materials

**Figure 1:**
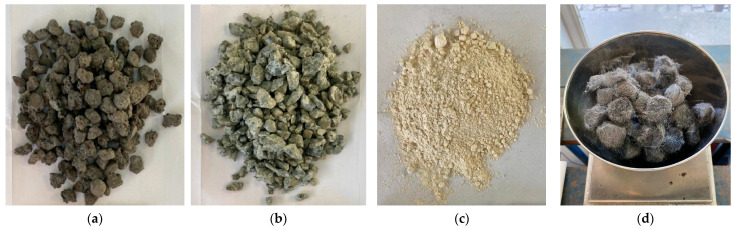
Different materials used in this study: (**a**) steel slag aggregates, (**b**) natural granite aggregates, (**c**) limestone filler, and (**d**) steel wool fibers.

The materials used in this study were a 35/50 pen-grade bitumen and those presented in Figure 1, which include steel slag aggregates (SSA), natural granitic aggregates (NGA), limestone filler (LF), and steel wool fibers (SWF).

### 3.2. Aggregates and Bitumen Characterization

This study used natural granite and steel slag aggregates formerly characterized at the University of Minho, as presented in a previous study [10]. In that study, several tests indicated the aggregates’ properties, including the particle density and water absorption, the wear resistance through the micro-Deval coefficient, the resistance of coarse aggregates to polishing, and the binder–aggregate affinity. The authors concluded that SSA has excellent potential for incorporation into asphalt mixtures due to its excellent wear and polishing resistance and good bitumen affinity.

The particle size distribution of the different aggregate fractions for the SSA (0/10 and 10/14) and the natural aggregate fractions (0/4, 4/6, and 6/14) was determined using the EN 933-1 standard. Those results were essential to designing asphalt mixtures that comply with the grading envelope of the Portuguese specifications [25].

The bitumen used in this study was characterized by the penetration test at 25 °C, according to EN 1426, and the softening point test using the ring and ball method, according to EN 1427.

### 3.3. Definition of the Percentage of Steel Wool Fibers

The percentage and geometry of the fibers selected in this work to incorporate in the asphalt mixtures for self-healing assessment were based on previous studies.

Liu et al.’s [26] study used a fiber percentage of 6% of the bitumen volume with positive results since the asphalt mixtures showed higher strength values than those without fibers. In the same paper, the authors mentioned the excellent resistance to particle loss and acceptable induction speed of the mixtures with fibers. However, the fiber length was only 4.2 mm, which was not ideal for a faster temperature rise in asphalt mixtures.

Tabaković et al. [27] used three different fiber percentages for each asphalt mixture (5, 10, and 15% of the bitumen volume) with a fiber length of 10 mm. The asphalt mixture with 5% fibers had several advantages, showing the best self-healing results. Moreover, the good results were visible to the naked eye since the crack closed entirely after healing, and the asphalt mixture did not present additional problems, such as, for example, the formation of crusts or oxidation, observed in mixtures with a higher percentage of fibers.

Gallego et al. [28] produced several asphalt mixtures with different percentages of fibers between 0.2% and 1.8% of the mixture’s total mass, using fibers with a variable length (5 mm or 10 mm) and diameter (0.04–0.06 mm and 0.10–0.12 mm) in each mixture. That paper demonstrated that the energy efficiency of the 10 mm-long fibers is higher than that of the 5 mm-long fibers, and the fibers with 0.04–0.06 mm thicknesses had the best healing performance. Finally, the authors confirmed that only 0.2% of the fibers over the mixture’s total mass was sufficient to obtain the expected performance for 10 mm-long fibers.

Therefore, based on the mentioned articles, this work selected the following conditions to produce mixtures B and C with steel wood fibers: a percentage of fibers equal to 7% of bitumen mass or 0.35% of the asphalt mixture total mass, with lengths of nearly 10 mm and a diameter between 0.04 and 0.06 mm. The fibers were cut from steel wool into pieces with the chosen lengths and then separated manually to obtain the necessary amount of fiber.

### 3.4. Mix Design, Production, and Characterization of Asphalt Mixtures

The following asphalt mixtures (AC 14 surf 35/50) were selected for this study to evaluate the composition influence on their mechanical and self-healing performance:

- Mixture A (MA)—a conventional mixture with natural aggregates.
- Mixture B (MB)—a new mixture with natural aggregates and steel wool fibers.
- Mixture C (MC)—a new mixture with steel slag aggregates, natural aggregates, and steel wool fibers.

The asphalt mixtures design was based on the various aggregate fractions’ density and particle size distribution, adjusting the final grading curves within the AC 14 surf grading envelope stated in the Portuguese standards [25]. Mixture C incorporated 50% steel slag aggregates, while mixtures A and B were produced with natural aggregates.

In this study, a 5.0% bitumen content was defined for all mixtures to reduce the number of independent variables under analysis. The bitumen content was defined based on the optimum content determined in a previous study through the Marshall mix design method [10]. Steel wool fibers (SWF) were added to the bitumen and aggregate blend for mixtures B and C. The mass of steel wool fibers added was the same for both mixtures, i.e., 7% of the bitumen mass of each mixture. The mixtures’ production occurred at a mixing temperature of 165 °C, specified in EN 12697-35 standard, after heating the aggregates at 170 °C and the bitumen at 165 °C. The materials of each mixture were mixed roughly for two minutes until the aggregates were fully covered by bitumen. In mixtures B and C, the steel wool fibers were gradually placed into the mixer bowl during the total mixing time to distribute the fibers better.

Two pieces of equipment were used for asphalt mixture compaction. First, three rectangular slabs of each mixture were compacted at 150 °C with the roller compactor equipment, according to EN 12697-33. This compaction uses a segmented roller with alternated operated rotation, simulating a street roller’s on-site action. The slabs were later cut into twelve beams (380 × 51 × 64 mm^3^). Then, nine cylindrical specimens of each mixture (diameter of 101 mm and a height of nearly 63 mm) were compacted at around 150 °C, applying 75 blows per side using a Marshall impact hammer, according to EN 12697-30.

The cylindrical specimens were initially used to study their microwave induction heating and, after being cut diametrically, to study the fracture toughness in a semicircular bending (SCB) test. The beams were tested for induction heating and were essential to determining the fatigue resistance and stiffness modulus in a four-point bending test.

After compaction, the samples’ bulk density (BD) was determined according to method B of EN 12697-6, and the mixture’s maximum density (MD) was assessed according to method A of EN 12697-5. Both values were used to calculate the air voids content of each asphalt mixture, which must be controlled within the limits specified in the Portuguese specifications [25]. According to those specifications, AC 14 surface mixtures should have an air voids content between 3% and 5%. Furthermore, it is stated that the air voids content must be calculated using EN 12697-8 and EN 12698-6 standards.

### 3.5. Preliminary Microwave Induction Heating Tests of Asphalt Mixtures

Three cylindrical specimens of each mixture were used in a preliminary study to evaluate the thermal induction behavior of the different asphalt mixtures as a function of the microwave power and heating time. Several scientific studies analyzed different procedures for heating asphalt mixtures with microwave radiation. The ideal self-healing induction time varies depending on the equipment, specimen geometry, asphalt mixture type, and induction power [26,27,28].

**Figure 2:**
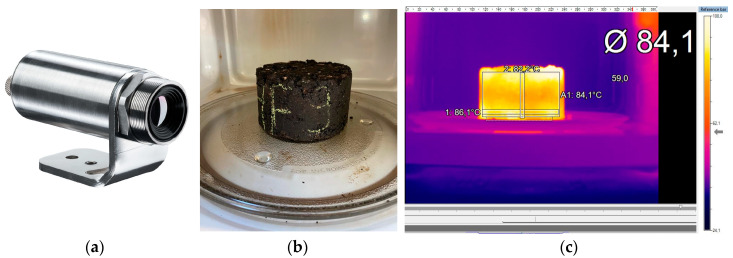
Procedure for determining the surface temperature of specimens: (**a**) thermographic camera, (**b**) asphalt specimen in the microwave oven, and (**c**) thermal image of the specimen.

Therefore, a preliminary analysis was performed in this work to assess the temperature evolution of the specimens when induced at different microwave power levels of 540 W, 720 W, and 900 W, with temperature measurements with a thermographic camera every 40 s for a total heating time of 160 s. Figure 2 shows the main steps for measuring the surface temperature of each test specimen. Before being tested, the specimens were placed in a thermal chamber at 20 °C to homogenize the initial test conditions. In addition, the results of the thermographic camera were previously calibrated in the laboratory.

The microwave radiation distribution was uniform over the surface of the cylindrical specimen since a rotating plate was used during the microwave heating. On the other hand, the microwave rotating plate could not be used when heating the fatigue test beams (Section 3.6.3) due to their dimensions. Thus, radiation may have been unevenly distributed over the whole surface of the beams.

### 3.6. Self-Healing Assessment and Mechanical Characterization of the Asphalt Mixtures

Mechanical characterization is essential to analyzing the performance of any asphalt mixture, but it gained additional importance in this work. Some properties of the studied asphalt mixtures (e.g., fracture strength and fatigue life) were assessed at different damage stages to calculate the healing ratio derived from a microwave thermal induction process. Thus, the self-healing performance of the studied asphalt mixtures was assessed by a cyclic set of mechanical tests (i.e., semicircular bending, fatigue, and stiffness modulus tests) followed by microwave radiation heating periods applied to heal the damaged specimens, as detailed in the following sections.

#### 3.6.1. Semicircular Bending Test

The asphalt mixtures were initially characterized through a three-point bending test performed on semicircular specimens, according to EN 12697-44 standard. This test evaluated the fracture toughness of the three asphalt mixtures, measuring the maximum fracture load and the corresponding displacement for the twelve specimens tested.

**Figure 3:**
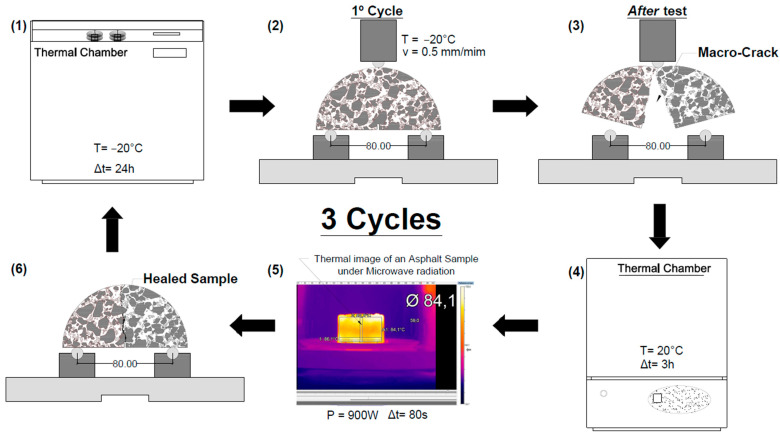
Cyclic setup of semicircular tests and microwave heating processes applied to assess the healing ratio.

The test was performed in a Matest Unitronic universal loading structure in a bending test configuration with two support points spaced out 8 cm apart and a load application point at the specimen’s midpoint, as presented in Figure 3. The load application speed was 0.5 mm/min, and the test temperature was −20 °C. This low temperature ensures a brittle fracture of the specimens, creating well-defined cracks that are easier to control during the healing phase. Before the fracture test, a 1 cm-deep notch was made at the semicircular specimens’ half span to regulate the crack’s start and progression.

The procedure adopted to calculate the healing capacity of asphalt mixtures through cycles of semicircular bending tests (fracture stage) and microwave radiation heating (healing stage) is summarized in the following steps and Figure 3.

1. The semicircular specimens are conditioned in a thermal chamber at −20 °C for 24 h.
2. The semicircular bending (SCB) test is performed at a speed of 0.5 mm/min.
3. The damaged specimens are placed in a ventilated thermal chamber at 20 °C for three hours to evaporate some moisture in the crack.
4. Microwave radiation heating is applied for 80 s using a microwave oven at a power of 900 W during the healing phase.
5. After induction, the specimens rest at room temperature for 2 h before repeating procedures 1 to 5 for three consecutive cycles.

The main objective of the fracture test was to determine the strength recovery (healing) capacity of the mixtures induced by heating after the complete rupture of the specimens. The average strength recovery was computed as the mixture healing ratio (HR), according to Equation (1). This parameter calculates the proportion between the peak load reached by the intact specimen before the healing cycles (F_initial_) and the peak load achieved by the damaged sample after each induction heating cycle of the healing process (F_final_).

(1) $$\mathrm{HR}=\frac{F_{\mathrm{final}}}{F_{\mathrm{initial}}}$$

Finally, after concluding the three healing cycles in this test configuration, the healing ratios of the three asphalt mixtures resulting from each healing stage were calculated to analyze the influence of each asphalt mixture composition on its healing capacity.

#### 3.6.2. Stiffness Modulus Test

The stiffness modulus is a fundamental characteristic of asphalt mixtures used as input in road pavement design and is essential to assessing the structural behavior of infrastructures with asphalt layers. This property depends on several factors, such as (1) temperature, (2) loading frequency, and (3) material characteristics.

The analysis of this property is complex from the point of view of the structural performance of a pavement. For example, increasing the stiffness increases the structure’s bearing capacity by reducing the resulting strain levels in the pavement layers. Nevertheless, asphalt mixtures with increased stiffness can also reduce the flexibility of the pavement, reducing fatigue cracking resistance.

Three beams of each mixture were tested in a four-point sinusoidal bending test configuration with two support points at the ends and two load application points in the middle span of the beam to evaluate the stiffness modulus of the studied asphalt mixtures. The test was performed under controlled strain at 20 °C according to the EN 13108-20 standard. In addition, a low peak-to-peak strain level (1 × 10^−4^) was set to assess the stiffness modulus in the linear region of the asphalt mixtures’ viscoelastic behavior. In this test, the amplitude of the sinusoidal strain wave was plus-minus 50 microstrains. A frequency sweep test (0.1, 0.2, 0.5, 1, 2, 5, 8, and 10 Hz) was carried out according to EN 12697-26 standard procedures to determine the stiffness modulus of asphalt mixtures.

Those three beams were tested twice again after being exposed to two healing cycles to determine the change in the stiffness modulus caused by induction heating with a microwave (210 s at a power level of 900 W).

#### 3.6.3. Fatigue Cracking Test

The fatigue cracking test evaluates the behavior of asphalt mixtures concerning fatigue cracking caused by repetitive loads of heavy traffic under specific weather conditions. This test is performed following the EN 12697-24 standard, applying a sinusoidal cyclic load at a frequency of 10 Hz in a four-point bending configuration (similar to the stiffness modulus test) at 20 °C, as suggested by the EN 13108-1 standard. Unlike the stiffness modulus test, the fatigue test is destructive since it applies high strain levels (4 × 10^−4^) that cause progressive fatigue cracking damage to the nine asphalt mixture beams tested.

The fatigue cracking test aims to determine the fatigue life of the mixtures, i.e., the number of cycles required for the asphalt mixture to reach fatigue failure. The number of load cycles at which the stiffness modulus reduces to half its initial value (N_0.5_) is usually the fatigue failure criterion used to define the fatigue life of the beams for each strain level when fatigue tests are performed under strain control. However, induced healing of road pavements should be used as a maintenance technique and is not expected to be only applied after total fatigue cracking failure. Thus, other approaches were explored in the literature to define a less aggressive fatigue-stopping criterion, allowing an improved healing performance of the asphalt mixtures during their thermal induction in the microwave.

During the literature review, several papers advocated that the beams should not be tested until total fatigue cracking failure to obtain a better healing ratio. Garcia et al. [29] showed that the optimum moment to induce self-healing of asphalt mixtures occurs at 35% of the number of cycles of total fatigue failure (0.35 × N_0.5_). Liu et al. [30] presented positive self-healing results using a more practical approach to define the fatigue failure criterion before applying healing cycles. The authors set a new fatigue stop criterion as the number of load cycles causing a reduction of 35% in the initial stiffness modulus (N_0.35_).

**Figure 4:**
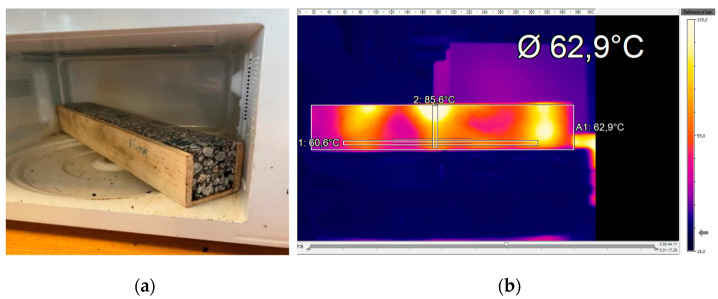
Procedure for determining the surface temperature of specimens: (**a**) asphalt beam inside a wooden mold in the microwave oven and (**b**) thermal image of the beam.

In this work, the fatigue tests considered the same fatigue failure criterion (N_0.35_) before applying each healing stage, stopping the fatigue test when the initial stiffness modulus was reduced by 35%. During the healing stage, the beams were put in the microwave oven (Figure 4) inside a wooden mold to prevent permanent deformation at high temperatures. The asphalt mixtures were then heated for 210 s at a power level of 900 W since these were the optimum conditions observed for beams. Finally, the beam’s temperature distribution was measured using a thermographic camera.

**Figure 5:**
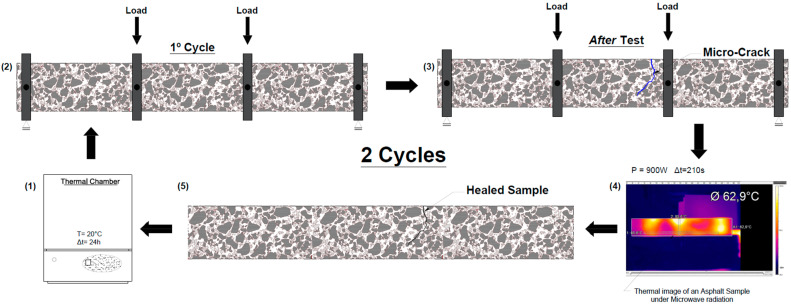
Cyclic setup of fatigue tests and microwave heating applied to assess the healing index.

After the first healing stage, the beams were tested again to evaluate asphalt mixtures’ fatigue life, using the same fatigue failure criterion (N_0.35_), before applying a second healing cycle. Finally, a third fatigue test was carried out on the healed beams to evaluate the fatigue life for two fatigue failure criteria: (1) N_0.35_ to calculate the healing index after fatigue and (2) N_0.5_ to assess the total fatigue failure of the mixtures. The cyclic setup of the fatigue tests and microwave heating stages is shown in Figure 5.

This cyclic setup evaluated the pavement’s service life gain after healing by comparing the fatigue life (number of cycles corresponding to N_0.35_) of beams subjected to fatigue tests before and after microwave radiation heating. The regeneration or healing index (HI) was calculated to determine the gain in a pavement’s service life [31].

After carrying out the consecutive fatigue tests, the HI value was computed as the ratio of the differential fatigue life after healing (𝑁_𝑓_ − 𝑁_𝑥_) to the fatigue life in the first test (𝑁_𝑥_), as shown in Equation (2), for a given fatigue failure criterion (35% reduction of the initial stiffness). 𝑁_𝑓_ is the fatigue life of the mixture after healing (no. of cycles), and 𝑁_𝑥_ is the mixture fatigue life in the first fatigue test (no. of cycles) before healing.

(2) $$\mathrm{HI}=\frac{N_{f}-N_{x}}{N_{x}}$$

## 4. Results and Discussion

### 4.1. Aggregates and Bitumen Characterization

The particle size distributions of the NGA and SSA fractions were obtained before designing the studied asphalt mixtures. The grading curves showed that the SSA material has fewer fines than the NGA, which increased the use of limestone filler in mixture C. The density values determined by Moura et al. [10] for these materials (2.65 Mg/m^3^ for NGA and LF; 3.25 Mg/m^3^ for SSA) were also used to design the asphalt mixtures.

The 35/50 pen-grade bitumen used in this work is the most widely applied in Portugal. The bitumen presented a penetration value of 44.9 tenths of a millimeter and a softening point temperature of 51.1 °C, and both values are within limits established by the EN 12591 standard for this type of bitumen.

### 4.2. Mix Design, Production, and Characterization of Asphalt Mixtures

Table 1 presents the compositions of the three asphalt mixtures (A, B, and C) studied in this work by showing the percentage volume of each aggregate fraction used. The corresponding mass values used to produce the mixtures were determined based on the aggregates’ densities. The composition was defined based on the particle size distribution of the different aggregates to obtain a final grading curve of each asphalt mixture fitting the grading envelope of an AC 14 surf defined in the Portuguese standards [25].

The asphalt binder content of mixtures A and B was 5.0%, using an equivalent value in mixture C, adjusted to 4.5%, to consider the higher density of the SSA (3.29 Mg/m^3^) in comparison to the NGA (2.64 Mg/m^3^). The percentage of steel wool fibers added to mixtures B and C was 7% of bitumen weight. The fibers were included as an additive and were not considered when adjusting the aggregate grading curve of those mixtures.

The volumetric characteristics of the asphalt mixtures were obtained by assessing their maximum density (MD) and bulk density (BD) of compacted specimens. Then, each specimen’s air voids content (Vv) was computed from MD and BD values. Table 2 presents the mean volumetric characteristics for each asphalt mixture.

The three mixtures have similar air voids contents, slightly below the limit defined in the specifications. The high density and low air voids content of mixture C should be emphasized from these results. Good workability was observed for the mixture with 50% SSA during the laboratory production and compaction phases, which solved the high air voids content issue mentioned in Moura et al.’s [10] study for a similar asphalt mixture.

### 4.3. Preliminary Microwave Induction Heating Tests of Asphalt Mixtures

**Figure 6:**
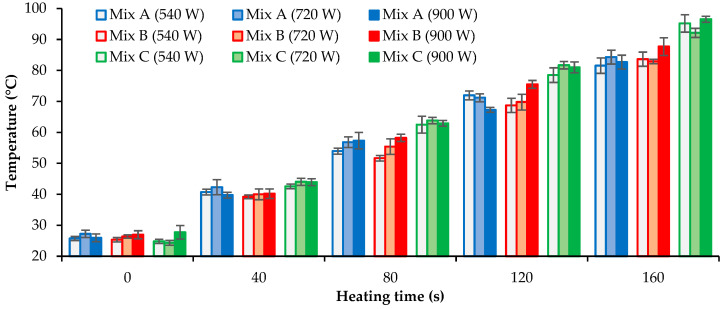
Evolution of surface temperature of the different asphalt mixtures with the heating time for three microwave power levels.

In the present work, the influence of the asphalt mixture composition was initially studied by assessing its thermal induction behavior when heated by microwave radiation. The temperature evolution of cylindrical specimens was evaluated every 40 s, for a total time of 160 s, with different microwave power levels (540 W, 720 W, and 900 W) to find differences in the heating rate between the studied mixtures. The tests were performed according to the procedures and techniques described previously. Figure 6 shows the mean surface temperature of three specimens of each asphalt mixture (A, B, and C) as a function of the heating time for the different microwave power levels used.

**Figure 7:**
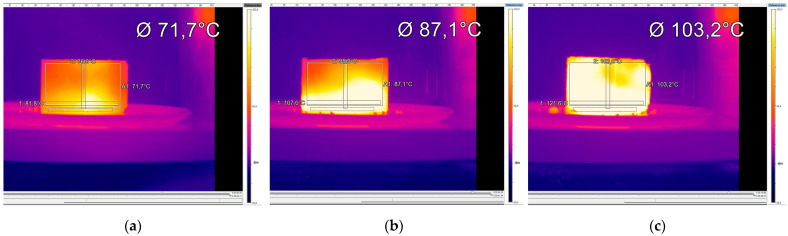
Thermal images of the different asphalt mixtures for the same radiation conditions (160 s and 900 W) in the microwave: (**a**) mixture A, (**b**) mixture B, and (**c**) mixture C.

These results show that mixture B generally presented slightly higher temperatures than mixture A for identical induction times. Mixture C showed higher temperatures for all induction periods and power levels, mainly for longer induction times (80 s to 160 s). Thus, the combined use of SSA and SWF increases the heating rate in the microwave induction process. The temperatures measured with the thermographic camera had a very low deviation, assuring high confidence in the obtained results. The positive influence of incorporating SSA and SWF (mixture C) becomes evident in the thermal images of Figure 7, obtained for the same microwave heating conditions (160 s and 900 W).

The influence of microwave power levels on the heating results was not as substantial as expected, probably due to the equipment used. However, temperatures evolved more rapidly and uniformly for the 900 W power, particularly in mixtures B and C. Thus, that power level was selected for this study’s remaining microwave heating tests. Long induction times (i.e., higher than 120 s) should be avoided when heating cylindrical specimens to reduce bitumen aging at high induction temperatures (near 100 °C) since bitumen becomes stiff and brittle, decreasing the fatigue and fracture resistance of the mixtures.

### 4.4. Self-Healing Assessment and Mechanical Characterization of the Asphalt Mixtures

This section mainly discusses the self-healing performance of the studied asphalt mixtures resulting from their fracture recovery or fatigue life increase after successive cycles of mechanical tests (damaging stage) and microwave heating (healing stage).

#### 4.4.1. Cyclic Setup for Semicircular Bending Tests and Microwave Heating

**Figure 8:**
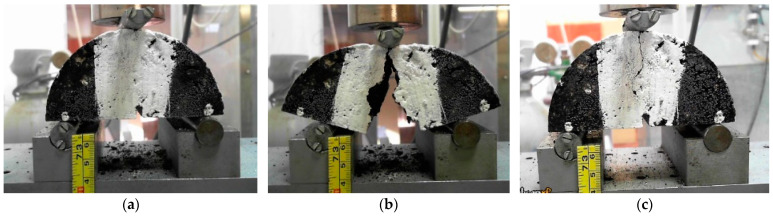
Asphalt mixture specimen: (**a**) before the SCB test; (**b**) damaged after the fracture test; (**c**) after microwave healing.

In this study, a three-point semicircular bending test (Figure 8a) was performed to evaluate the fracture toughness of the different asphalt mixtures and their ability to recover (Figure 8c) their mechanical performance after total failure (Figure 8b). The macro-cracks observed in fracture tests may have inhibited the self-healing action compared to micro-cracks occurring in fatigue tests, but this work assumed that challenge.

The experimental procedure used thirty-six semicircular specimens (twelve of each mixture) that underwent several testing cycles, with four SCB fracture test repetitions, before and after three healing cycles of induction heating via microwave radiation (during 80 s at a power level of 900 W). An elastic band wrapped the specimens to facilitate the healing of the fractured faces of the semicircular samples during the induction period.

Table 3 presents the mean results of the four SCB fracture test repetitions performed over the three healing cycles for the studied asphalt mixtures.

**Figure 9:**
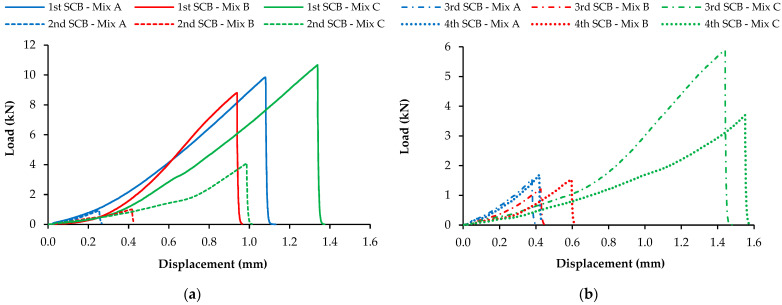
Examples of load vs. displacement evolution of the different asphalt mixtures during the four fracture test repetitions: (**a**) 1st and 2nd SCB tests; (**b**) 3rd and 4th SCB tests.

After collecting and organizing the data (load and displacement) from the semicircular bending fracture tests, the most relevant results for each specimen, the maximum load and the peak displacement, were analyzed. The load vs. displacement graphs of the four SCB test repetitions were plotted for all the tested specimens. However, Figure 9 presents examples of only one characteristic sample of each asphalt mixture studied during this stage since showing all the results would be inviable.

**Figure 10:**
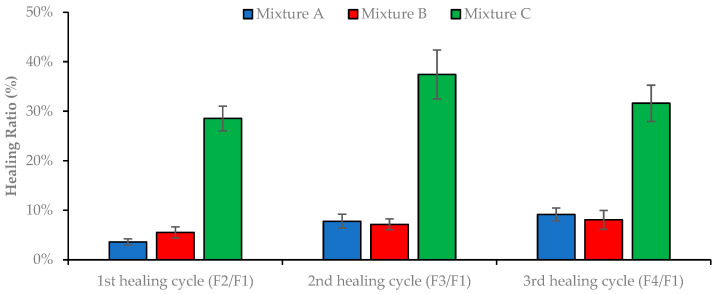
Healing ratio values of the semicircular test specimens after the three healing cycles.

The healing ratio (HR) value was calculated as the ratio of the peak load of the specimens after each healing cycle to the corresponding peak load of the intact asphalt mixture in the first SCB test. The HR values of the three asphalt mixtures after each healing process are presented in Figure 10. When analyzing the results, it should be noted that the semicircular specimens presented brittle behavior in the SCB fracture test, as expected, due to the low temperature (−20 °C) used.

Based on the data obtained, it is noticeable that the fracture damage caused by the SCB test recovered more significantly after healing in mixture C. This mixture recovered about 40% of its initial fracture toughness for increased peak displacements at rupture in all the SCB test repetitions performed after healing. Thus, multiple healing stages may be applied for road pavement maintenance. Nevertheless, the other asphalt mixtures (A and B) showed lower healing ratios (around 10%) after total damage.

Due to the destructive nature of SCB tests, the results of this test presented a higher deviation than other tests carried out in this work. A null hypothesis one-way ANOVA (alpha level of 0.05) compared the healing ratio of the three asphalt mixtures considering the three healing cycles, showing a statistically significant difference in the mean HR between at least two asphalt mixtures (F(2, 33) = 33.077 to 76.442, and *p* = 0.000). The Tukey’s HSD test for multiple comparisons found that the HR was significantly different between mixtures A and C (*p* = 0.000, 95% C.I. = [16.02%, 33.33%] after the third healing cycle) and mixtures B and C (*p* = 0.000, 95% C.I. = [16.35%, 33.66%] after the third healing cycle). On the other hand, the mean healing ratios of mixtures A and B are similar (*p* = 0.995).

**Figure 11:**
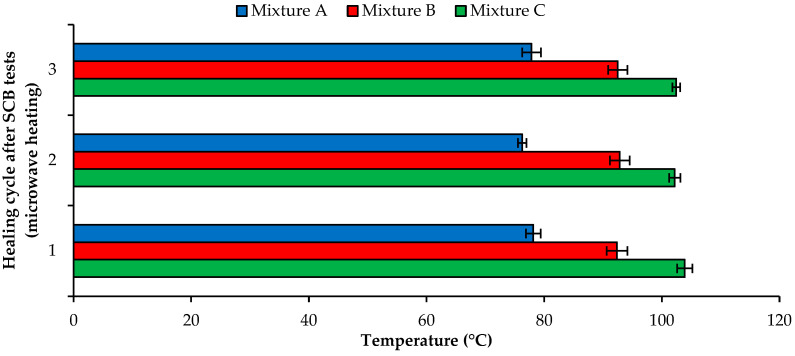
Mean temperatures of the mixtures induced by microwave heating in each healing cycle.

Finally, Figure 11 shows the mean temperatures of the three asphalt mixtures achieved during microwave heating (900 W power and 80 s heating time) of the semicircular specimens for each healing cycle between the SCB tests.

The results obtained at this stage align with those previously presented in the preliminary microwave heating tests. Once again, mixture C (combining SSA and SWF) showed a faster temperature growth for the same heating conditions, which may justify the best self-healing performance of this mixture after microwave heating. Moreover, the conductive materials (steel slag and steel fibers) used in mixtures B and C increased the microwave heating efficiency compared to conventional asphalt mixture A. The range of temperatures measured for different mixtures after 80 s of microwave radiation is very high (30 °C), demonstrating the importance of using conductive materials to improve heating. A similar one-way ANOVA assessment compared the microwave heating efficiency when heating the three asphalt mixtures considering the three healing cycles, clearly showing a statistically significant difference in the mean temperatures achieved by all asphalt mixtures (F(2, 33) = 78.233 to 123.876, and *p* = 0.000). Further research should be developed to define induction times according to the expected healing temperature because this factor may influence self-healing performance.

#### 4.4.2. Cyclic Setup for Stiffness Modulus Tests and Microwave Heating

**Figure 12:**
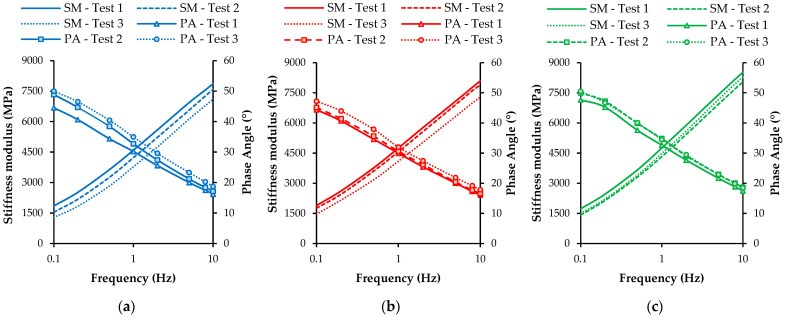
Evolution of the stiffness modulus (SM) and phase angle (PA) of the studied mixtures before healing (test 1) and after one (test 2) and two (test 3) healing cycles: (**a**) mixture A; (**b**) mixture B; (**c**) mixture C.

The stiffness modulus of the studied asphalt mixtures was measured based on the procedures previously presented. Figure 12 shows the evolution of the stiffness modulus and phase angle of mixtures A, B, and C with the frequency at 20 °C. Tests were made before healing (test 1) and after two healing cycles (tests 2 and 3) in the microwave.

The results of these tests show the variation of the asphalt mixtures’ stiffness modulus and phase angle due to the microwave heating process. The results demonstrated that the microwave heating applied to the asphalt mixtures during the healing stage did not significantly change the fundamental properties of the studied asphalt mixtures. Nevertheless, a slight reduction in the stiffness modulus can be observed for all asphalt mixtures, indicating that some structural damage from testing the asphalt mixtures was slightly more influential than the healing effect and bitumen aging (stiffening) during microwave heating.

**Figure 13:**
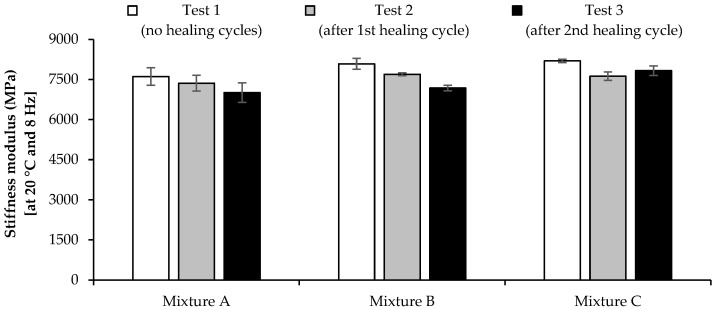
Stiffness modulus of the mixtures obtained in the test conditions mentioned in the standard (frequency of 8 Hz and temperature of 20 °C).

Figure 13 presents the variation of the stiffness modulus (i.e., the mean value of individual results for three beams of each asphalt mixture) at standard test conditions of 8 Hz and 20 °C before and after two healing cycles. A null hypothesis one-way ANOVA (alpha level of 0.05) compared the stiffness modulus of the three asphalt mixtures considering the different healing cycles, revealing that there is no statistically significant difference in the mean stiffness modulus between the three asphalt mixtures (F(2, 6) = 0.808 to 3.177, and *p* = 0.115 to 0.489). The healing process’s effect on the mixtures’ mean stiffness modulus was only significantly different for mixture B between tests 1 and 3 (*p* = 0.008), presenting *p* values lower than the alfa level of 0.05 in all other situations.

These results confirmed the low influence of microwave heating on the stiffness modulus of asphalt mixtures. However, it is possible to observe that mixture C slightly increased the stiffness modulus during the second healing cycle, probably due to the higher temperatures induced in mixture C by microwave heating, which may have improved the mixture healing and the binder–aggregate or binder–SSA bond while increasing bitumen aging.

#### 4.4.3. Cyclic Setup for Fatigue Tests and Microwave Heating

A four-point sinusoidal bending test was performed on the studied asphalt mixtures to evaluate their fatigue cracking resistance before and after two healing cycles. The failure criterion in the fatigue test was set for a stiffness reduction of 35% from its initial value to optimize strength recovery during the healing stages. The corresponding number of loading cycles was recorded as the beam’s fatigue life (N0.35). Then, after a healing stage with microwave heating (900 W for 210 s), the beams were tested again twice to evaluate the extended fatigue life (N_0.35_) using the same failure criterion. The ratio between the extended fatigue life after healing (in the two fatigue test repetitions) and the intact beams’ original fatigue life was defined as the mixtures’ fatigue life gain (healing index).

For this cyclic setup of fatigue tests and microwave heating, nine intact beams of each asphalt mixture were tested for fatigue (1st fatigue test), stopping the test after a stiffness reduction of 35% (N_0.35_). Then, the first stage of microwave heating was applied (1st healing cycle). The healed beams were tested again for fatigue (2nd fatigue test) using the same failure criterion (N_0.35_) just before the second microwave healing cycle. Finally, the beams were tested again for fatigue (3rd fatigue test) until total fatigue failure of the mixtures for a stiffness reduction of 50% (N_0.5_), but the fatigue life N_0.35_ was also registered.

Table 4 presents the results from the fatigue tests performed in this study. The results show a significant stiffness recovery of the fatigue cracking damage after each fatigue test and microwave healing stage for all mixtures. Thus, the fatigue failure criterion and thermal radiation process used to induce healing were adequately selected.

Moreover, the initial stiffness modulus (E_initial_) in the third fatigue test (after two healing cycles) was higher than before healing for all mixtures, probably due to bitumen aging.

**Figure 14:**
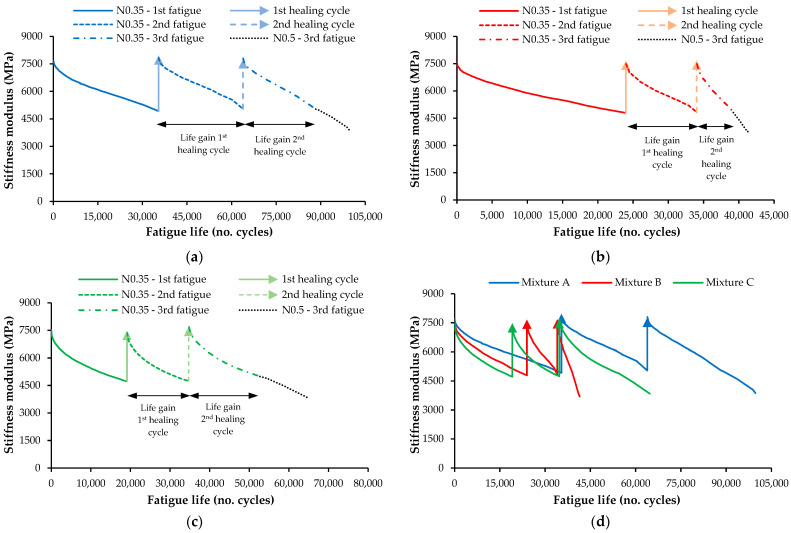
Evolution of the stiffness modulus during three fatigue tests performed before (intact beam) and after two healing cycles in the microwave: (**a**) mixture A; (**b**) mixture B; (**c**) mixture C; (**d**) comparison of all mixtures.

Figure 14 exemplifies the fatigue life gain of each asphalt mixture (A, B, and C) after two thermal healing cycles performed at a fatigue failure criterion of 35% reduction of the stiffness modulus. Figure 14d compares the behavior of the three asphalt mixtures.

After applying the two microwave radiation healing cycles, the results show a noticeable fatigue life gain of asphalt mixtures, particularly for mixtures A and C. Therefore, a similar heating process may be applied for preventive maintenance of road pavements. Such an approach may improve the self-healing of asphalt mixtures and significantly extend their fatigue life and the durability of the pavement. Mixtures A and C increased their fatigue lives by far more than 100%, demonstrating the expected advantages of this solution. Therefore, by using these innovative solutions in new asphalt pavements, the environmental and economic costs could be reduced compared with the current expenses of traditional conservation works. Nevertheless, during the first fatigue cycle (before healing), the conventional mixture performed better than the alternative mixtures with SWF and SSA, demonstrating the importance of carefully designing conductive asphalt mixtures to increase their mechanical performance.

Based on the data acquired from the fatigue tests (fatigue life, mean number of cycles for fatigue failure at 35% reduction of the initial stiffness, or N_0.35_) for the intact beams before healing (*N_x_*) and the healed asphalt beams after each healing cycle (*N_f_*), the healing index (HI) of the studied asphalt mixtures was computed with Equation (2). This study considered this HI value the most representative since it uses the same fatigue failure criterion (0.35 × E_initial_) before and after healing. Moreover, the data of the three fatigue test repetitions were obtained from the same beam for each asphalt mixture. Therefore, this index can verify the fatigue life gain (number of cycles) resulting from the self-healing properties of each asphalt mixture and enhanced by microwave heating radiation.

**Figure 15:**
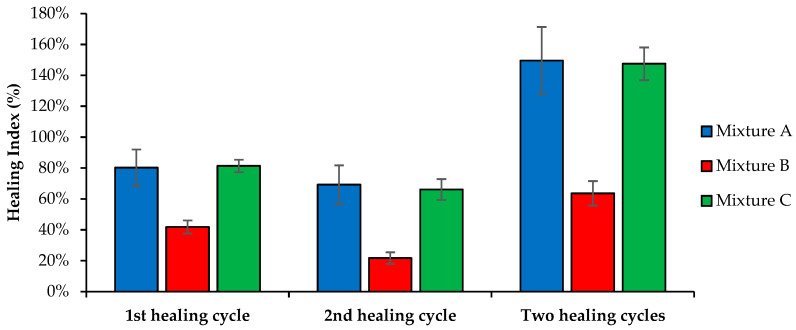
Healing index (HI) of each asphalt mixture in the fatigue test after two healing cycles.

Figure 15 shows the HI values of the three asphalt mixtures after isolating the extended fatigue life gain of the first and second healing stages or adding both cycles.

The fatigue life gain is perceptible for all studied mixtures from the data presented. Mixture A showed excellent results, with an HI or fatigue life gain of 149% due to the self-healing phenomena enhanced by the microwave radiation heating system. Thus, microwave heating also boosts the regeneration capacity of conventional asphalt mixtures. Mixture C (incorporating SSA and SWF) also showed excellent self-healing results since the fatigue life increased by 147% after two microwave healing cycles. Mixture B showed the worst HI value in this test but raised the fatigue life by around 65%. The mean errors obtained for the HI results confirmed the reliability of the conclusions obtained from this test, even though Mixture A presented a slightly higher dispersion of results.

A null hypothesis one-way ANOVA (alpha level of 0.05) was performed to compare the type of asphalt mixtures’ effect on the healing index after the different healing cycles, revealing that there was a statistically significant difference in the mean HI between at least two asphalt mixtures (F(2, 6) = 5.350, and *p* = 0.010 to 0.018) after healing. The Tukey’s HSD test for multiple comparisons found that the healing index was significantly different between mixtures A and B (*p* = 0.015 to 0.030, 95% C.I. = [21.37%, 149.29%] after two healing cycles) and mixtures B and C (*p* = 0.017 to 0.028, 95% C.I. = [19.37%, 147.29%] after two healing cycles). There was no statistically significant difference between the mean healing indexes of mixtures A and C (*p* = 0.960 to 0.995).

**Figure 16:**
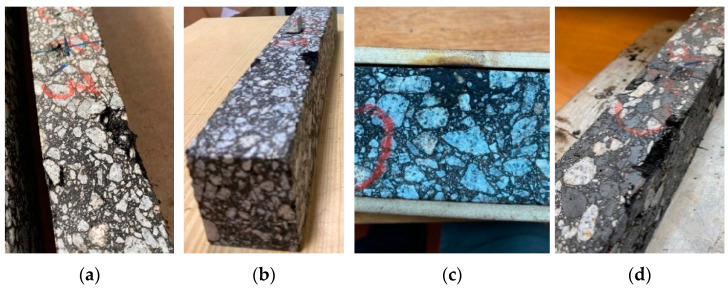
Sporadic degradation of beams after some cycles of fatigue tests and microwave heating. (**a**) Mixture A, (**b**) Mixture B, (**c**) Mixture B, (**d**) Mixture C.

The HI values decreased from the first to the second healing cycle in all mixtures, demonstrating some loss of efficiency when several healing repetitions are applied to recover fatigue cracking damage. Thus, further studies should be done to limit the number of healing cycles so that microwave radiation does not result in asphalt mixture performance problems. Figure 16 confirms that some beams experienced small deformations and crumbling in the load application zones of the fatigue test mechanism with the increasing number of fatigue tests and healing processes performed in this study.

## 5. Conclusions

This study mainly aimed to complement the current knowledge about asphalt mixtures self-healing using a microwave radiation heating method. New compositions for asphalt mixtures were studied using an industrial by-product, steel slag aggregates, and steel wool fibers.

The study’s results revealed that the combined use of these conductive materials in asphalt mixtures could enhance their heating induced by microwave radiation and improve the self-healing capacity of asphalt mixtures for future maintenance of road pavements. The following points summarize the main conclusions of this study:

- The healing capacity of the asphalt mixtures can be assessed through cycles of mechanical damage tests (i.e., fracture or fatigue) and microwave radiation heating (healing stage), as the specimens always recovered some strength after damage.
- The fracture tests on semicircular specimens of asphalt mixtures exhibited some strength recovery after healing induced by microwave heating. However, only mixture C (with SSA and SWF) showed sufficiently high healing ratios, around 40%, while the other two mixtures showed lower healing ratios of 10%.
- The cyclic setup of fatigue tests and microwave heating confirmed a significant fatigue life gain after healing (nearly 150% for mixtures A and C and 65% for mixture B).
- The number of microwave heating cycles applied to recover fatigue cracking damage should be limited due to some loss of efficiency after several healing repetitions.
- Combining steel slag aggregates and wool fibers in new asphalt mixtures can enhance their microwave radiation heating and self-healing performance.

The results of this work suggest that a similar heating process should be replicated and applied for preventive maintenance of road pavements to improve the self-healing of asphalt mixtures and extend their fatigue life and durability of the pavement. Incorporating new materials (e.g., SSA and SWF) in the asphalt mixtures may enhance self-healing performance, with environmental and economic advantages. Nevertheless, additional work should be carried out on this topic to further improve the healing performance of asphalt mixtures, particularly for in situ applications.

## Figures and Tables

**Table 1:** Volume percentages of the aggregates’ fractions used in the different asphalt mixtures.

Fraction	NGA 0/4	NGA 4/6	NGA 6/14	SSA 0/10	SSA 10/14	LF
Mixture A	40%	14%	42%	0%	0%	4%
Mixture B	40%	14%	42%	0%	0%	4%
Mixture C	27%	0%	17%	36%	14%	6%

**Table 2:** Volumetric characterization of asphalt mixtures.

Property	Maximum Density (Mg/m^3^)	Bulk Density (Mg/m^3^)	Air Voids Content (%)
Mixture A	2.484	2.415	2.8
Mixture B	2.481	2.410	2.9
Mixture C	2.830	2.753	2.7

**Table 3:** Data and mean results of the four SCB tests performed to evaluate the healing ratio.

Variable		Mixture A		Mixture B		Mixture C	
		Mean	SD	Mean	SD	Mean	SD
1st SCB test (intact, before healing)	F (kN)	9.8	0.6	10.2	1.2	9.7	2.1
	δ (mm)	1.0	0.3	1.2	0.2	1.3	0.2
2nd SCB test (after 1st healing cycle)	F (kN)	0.4	0.2	0.6	0.4	2.8	0.9
	δ (mm)	0.2	0.1	0.3	0.2	0.8	0.3
3rd SCB test (after 2nd healing cycle)	F (kN)	0.8	0.4	0.7	0.3	3.6	1.1
	δ (mm)	0.3	0.1	0.3	0.1	1.1	0.1
4th SCB test (after 3^rd^ healing cycle)	F (kN)	0.9	0.5	0.8	0.6	3.1	0.6
	δ (mm)	0.3	0.1	0.3	0.2	1.0	0.4

**Table 4:** Average fatigue resistance of the studied mixtures before and after two healing cycles.

Mixture	Before Healing		After One Healing Cycle		After Two Healing Cycles		
	E_initial_ (MPa)	N_0.35_ (cycles)	E_initial_ (MPa)	N_0.35_ (cycles)	E_initial_ (MPa)	N_0.35_ (cycles)	N_0.5_ (cycles)
A	7568	35,414	7757	28,423	7748	24,521	35,838
B	7370	23,971	7414	10,038	7392	5218	7439
C	7267	19,125	7294	15,563	7681	12,647	19,808

## Data Availability

Data sharing does not apply to this article.

## Abbreviations

The following abbreviations are used in this manuscript:

BD	Bulk density
E_initial_	Initial stiffness modulus of the asphalt mixture in the fatigue test
HI	Healing index
HR	Healing ratio
LF	Limestone filler
MD	Maximum density
NGA	Natural granitic aggregates
PA	Phase angle
SCB	Semicircular bending
SD	Standard deviation
SM	Stiffness modulus
SSA	Steel slag aggregates
SWF	Steel wool fibers
Vv	Air voids content
